# Supplementary material for: Saccharomyces boulardii (CNCM I-745) improves intestinal damage in sepsis by remodeling intestinal flora structure
Source: Front Cell Infect Microbiol. 2025 Jul 31;15:1602792. doi: 10.3389/fcimb.2025.1602792 (PMC12350329; doi:10.3389/fcimb.2025.1602792)
Supplement: Supplementary file 1 [file DataSheet1.zip › summary/summary/7_Differential_analysis/2_LEfSe_result/Group_DW4_vs_DW0_0.05_3.0/LEfSe_Group_DW4_vs_DW0.cladogram.pdf]

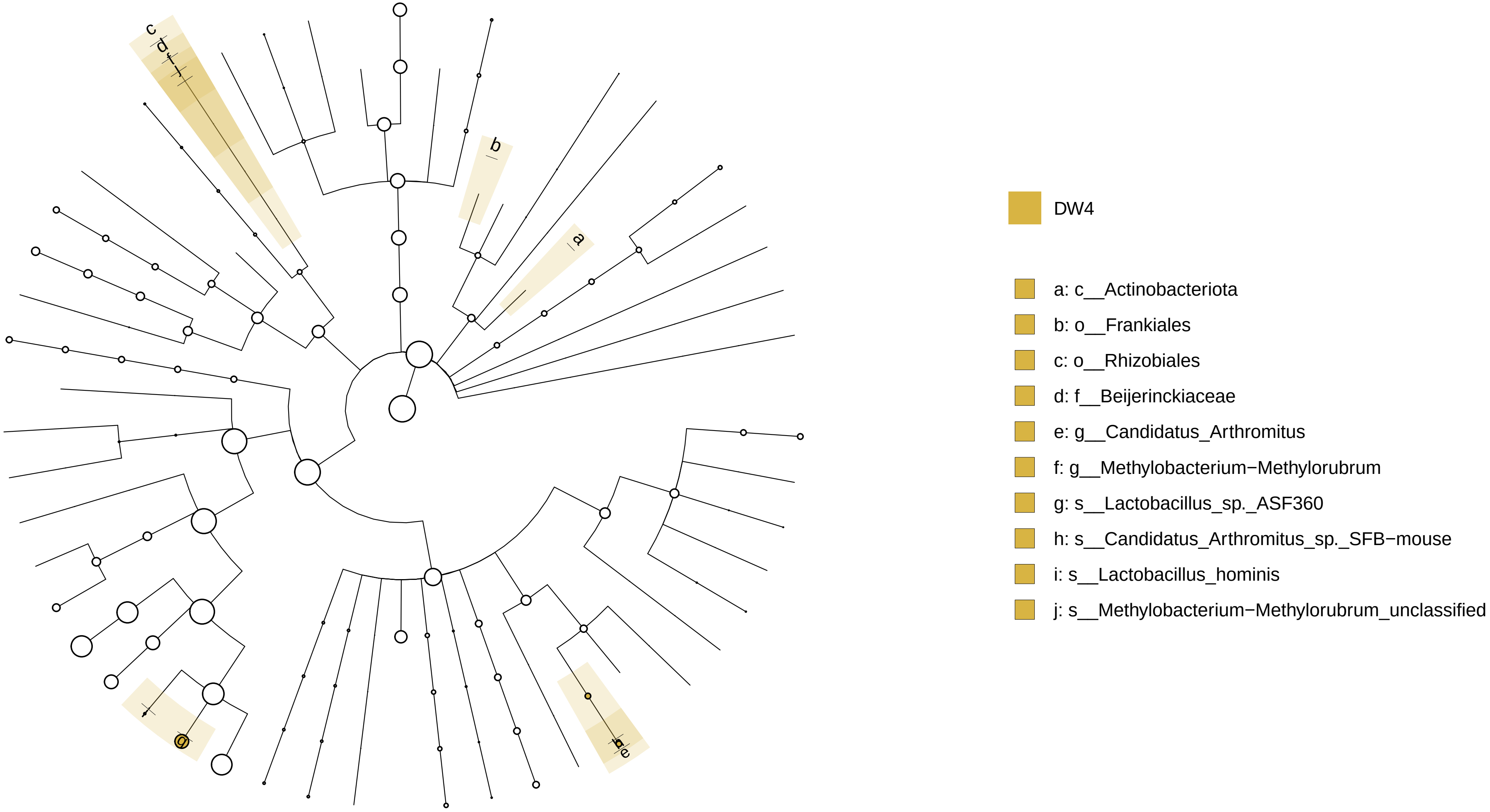

DW4
a: c__Actinobacteriota
b: o__Frankiales
c: o__Rhizobiales
d: f__Beijerinckiaceae
e: g__Candidatus_Arthromitus
f: g__Methylobacterium−Methylorubrum
g: s__Lactobacillus_sp._ASF360
h: s__Candidatus_Arthromitus_sp._SFB−mouse
i: s__Lactobacillus_hominis
j: s__Methylobacterium−Methylorubrum_unclassified
